# The Role of Coping Strategies in Post-Traumatic Growth among Syrian Refugees: A Structural Equation Model

**DOI:** 10.3390/ijerph18168829

**Published:** 2021-08-21

**Authors:** Busra Acar, İbrahim H. Acar, Omar A. Alhiraki, Ola Fahham, Yesim Erim, Ceren Acarturk

**Affiliations:** 1 Department of Psychology, Koç University, 34450 Istanbul, Turkey; bacar20@ku.edu.tr; 2 Department of Psychology, Ozyegin University, 34794 Istanbul, Turkey; ibrahim.acar@ozyegin.edu.tr; 3 Department of Surgery, Bab Al-Hawa Hospital, Idlib, Syria; dr.omar.alhiraki@gmail.com (O.A.A.); olafm2013@gmail.com (O.F.); 4 Department of Psychosomatic Medicine and Psychotherapy, University Hospital of Erlangen, Friedrich-Alexander University Erlangen-Nürnberg (FAU), 91054 Erlangen, Germany; Yesim.Erim@uk-erlangen.de

**Keywords:** post-traumatic growth, refugees, coping, post-migration stressors, Syrian conflict

## Abstract

The Syrian conflict has led to a mass migration of Syrians to other countries and exposed them to many possible traumatic events and stressors in their country of origin and in the resettlement process. The possibility of positive psychological effects of adverse life events is less documented among Syrian refugees. Thus, the current study aimed to develop preliminary evidence for the identifying factors: traumatic experiences, post-migration stressors and coping strategies that are associated with post-traumatic growth (PTG) of Syrian refugees residing in Turkey. Structural equation modeling (SEM) was used in the current study to assess the associations among these factors. Data were obtained from Syrian refugees residing in the governorates of Hatay and Mardin. A total of 528 Syrians, aged between 18–77 years (M = 35.60, SD = 11.65) participated in this cross-sectional study. Results from the SEM indicated that past traumatic experiences and post-migration stressors were indirectly related to PTG. The results from the current study provide support for that the association between refugees’ traumatic experiences, post-migration stressors and PTG appear to be explained through the presence of coping strategies which could be addressed in the psychotherapies and psychosocial interventions for refugees to promote positive psychological change. Future studies should address the effects of post-migration stressors on PTG in detail.

## 1. Introduction

According to United Nations High Commission on Refugees (UNCHR), there were 80 million forcibly displaced people worldwide in mid-2020, including 26.3 million refugees. The majority of refugees under the UNCHR mandatory come from the Syrian Arab Republic. Turkey hosts the largest number of registered refugees, with currently 3.6 million Syrian people affected by war, persecution, and armed conflicts [1].

The Syrian civil war exposed refugees to various possible traumatic events and stressors in their countries of origin, during flight and resettlement. Previous studies showed that most Syrian refugees have experienced several traumatic events such as witnessing the death of family members or friends, torture, and physical or sexual assaults in their country of origin [2,3]. During the flight, refugees could have additional overwhelming experiences such as walking on dangerous roads, traveling with unsafe boats, physical and sexual violence, infectious diseases, insufficient medical help, food, and clean water, and/or ending up in refugee camps [4,5,6,7]. Finally, the resettlement process has various difficulties which could be examined in three main areas, such as socio-economic, social/cultural, and refugee policies. Many struggles with finding employment, meeting basic needs such as food, shelter, healthcare, and education are often occurring during the resettlement process. Due to traumatic experiences and post-displacement related difficulties, they suffered from several psychological problems such as depression, anxiety, and trauma-related stress symptoms [8,9,10,11,12]. Despite the negative consequences of traumatic events, some refugees also report the subjective experience of positive psychological changes in response to highly stressful events, referred to as post-traumatic growth [3,13,14,15].

Post-traumatic growth (PTG) is defined as “positive psychological change experienced as a result of the struggle with highly challenging life circumstances” by Tedeschi and Calhoun [16] (p. 6). Positive changes can be identified: (1) improved closeness in relationships, (2) awareness of new possibilities in life (3) a sense of increasing personal strength, (4) positive spiritual change, and (5) increased appreciation of life. These psychological changes have been observed among several populations such as refugees and immigrants [17,18,19,20], civilians exposed to terrorism [21], survivors of interpersonal violence, and war veterans [22,23]. Limited literature has investigated PTG among Syrian refugees [14,24,25], and even less research has focused on the pathways of experiencing positive changes among them.

According to the PTG model of Tedeschi and Calhoun [16], a traumatic event that severely challenges and shatters the individual’s assumptions and beliefs about the world does not directly cause growth, but the emotional struggle following trauma is crucial for PTG to be experienced [16]. The association between trauma types and psychological growth was unexplained by this model. On the other hand, previous studies reported that trauma characteristics such as trauma types, number of traumatic events, and severity affect the level of PTG [26,27,28]. For instance, interpersonal negative events such as rape and torture were related to less growth [2] compared with non-interpersonal events such as natural disasters and accidents [29]. However, several studies showed that regardless of trauma characteristics, people might experience a similar level of PTG [30,31]. In addition to trauma type, previous studies indicated that people with multiple trauma exposure across their lifespan have reported more PTG [32,33]. As the abovementioned studies stated that refugees have encountered multiple traumatic events across their life span, it might be concluded that multiple trauma experiences were positively associated with PTG among refugees [18,34,35]. These findings might be explained by the severity of trauma that influences the emotional struggle to facilitate PTG. Previous studies reported that there was a positive correlation between PTG and the severity of exposure to past traumatic experiences [17,18,32,33]. Further, a study by Hussain and Bhushan [18] revealed that higher exposure to past traumatic experiences was positively associated with PTG among Tibetan refugees. In this regard, the dose–response relationship which posits an association between event magnitude and the clinical outcome could explain to which extent individuals experience growth following different and multiple traumas.

Post-migration difficulties could be seen as another type of trauma that leads to multiple social and emotional loss [36]. Despite being closely related, there is a growing consensus that post-migration difficulties and past traumatic experiences are conceptually distinct constructs [12,37] such that post-migration difficulties refer to the potential stressors subjectively experienced by refugees during the resettlement process [37]. On that ground, researchers have utilized these two constructs as separate entities in their models (e.g., [12,38,39]). Aligned with this conceptualization, we also used these two constructs as distinct variables. Limited studies have focused on the association between post-migration stressors such as acculturation problems, unemployment, social isolation, discrimination, and post-traumatic growth. Teoderescu et al. [3] reported that there was a moderate negative association between post-migration stressors and the level of PTG. On the other hand, in line with the dose–response relationship hypothesis, post-migration stressors might lead to accumulating stress level and, thereby, the high severity of trauma with cumulative nature might be positively associated with PTG among refugees [22,31]. Therefore, the resettlement process might have an effect on positive psychological change and growth. The controversial association between post-migration stressors and PTG could be explained by psychosocial factors such as coping strategies [40,41]. However, there has been a paucity of research focusing on growth among refugees who have been exposed to post-migration difficulties. As Lindencrone and colleagues [3] stated, comprehensive models that aim to identify potential risk and protective factors for refugees’ mental health must account for the post-migration difficulties. Therefore, in this current study, we will examine whether post-migration stressors positively or negatively contribute to growth via coping strategies.

Coping has been described as cognitive and behavioral efforts to handle external and/or internal demands that are deemed to be taxing or exceeding the person’s resources [42] (p. 141). Various classifications of people’s ways of coping exists in the literature, and they have been organized into higher-order categories (e.g., primary vs. secondary) [42]. In this sense, a widely used and accepted categorization of coping styles are problem-focused coping (dealing directly with stressor to remove it, e.g., planning), emotion-focused coping (dealing with the associated feeling distress by stressor, e.g., acceptance), and maladaptive coping styles (less useful, e.g., denial, substance abuse) [43]. Although problem-focused and emotion-focused coping strategies seem to have a similar vein in terms of adaptive ways of coping, previous studies showed that these two coping strategies have different functional ways and underlying mechanisms to handle stress [41,42]. Problem-focused coping focuses on actively addressing the problem itself, whereas the emotion-focused coping focuses on responses to emotions associated with the problem [44]. Further, researchers in coping literature posit that problem-focused coping strategies are more adaptive for the controllable situation, while emotion-focused coping strategies are more adaptive in uncontrollable situations [45,46]. On that ground, researchers have utilized these two constructs as separate entities in their models (e.g., [47,48]). Aligned with this conceptualization, we also used these two constructs as distinct variables. Adaptive and maladaptive coping strategies could be triggered by stressful life experiences and lead to how an event is perceived and responded to; thereby, these strategies might mediate the association between traumatic events and their outcomes [49]. In a similar vein, trauma and coping are considered to be related, and several studies indicated that trauma might diminish the capacity to cope with stressful life events [37] and increase the use of maladaptive coping strategies [50]; thereby, those coping strategies might be indirectly associated with trauma outcomes [18]. Furthermore, one of the previous studies reported that there is a negative correlation between past traumatic experiences and maladaptive and also adaptive coping strategies such as those that are problem-focused and emotion-focused [47]. Contrary to this finding, some studies indicated that traumatic experiences were positively associated with the use of emotion and problem-focused coping strategies [18,51]. Therefore, it is possible to conclude that the literature about the relationship between different coping strategies and past traumatic experiences is in part controversial.

In the Tedeschi and Calhoun model of PTG, coping is important to manage overwhelming emotions that arise from exposure to traumatic experiences, and it was found to be an important predictor of PTG [16]. Previous studies showed that problem-focused coping is positively correlated with PTG [27,41], as it can trigger rebuilding of assumptions of self, relations, and spiritual beliefs, and reflect the effort of reducing distress. Even though emotion-focused coping strategies could lead to focus on the negative side of traumatic experiences [52,53], emotional processing could facilitate meaning-making after negative life events [53]. Studies have shown that emotion-focused coping might enhance people’s understanding of the event; in turn, people might experience more growth [54]. On the other hand, avoidant coping (avoidance from related stressors or emotions) might be maladaptive in the long term and could be an obstacle for healing [55], but some studies have revealed that avoidant coping is positively related to PTG [56]. Thus, avoidant coping might play a role in the illusory coping process in PTG [57]. Further, acceptance coping was found to be positively correlated with PTG [58] while some studies reported no association between acceptance and PTG [53]. Although different coping strategies are significantly related to PTG, controversy remains about which coping style is more conducive to PTG. Researchers have emphasized that coping might be flexible across situations, and thereby ineffective strategies could change in response to particular life events [59]. Therefore, which coping strategies are used may depend on trauma characteristics and having a determinative role on the degree of PTG development.

Among different groups of refugees, past traumatic experiences and post-migration stressors might influence the use of coping strategies which, in turn, might have an effect on PTG [18,60]. A study by Hussain and Bhushan [18] found that cognitive coping strategies including positive refocusing, refocus on planning, putting into perspective, and catastrophizing partially mediated the relationship between past traumatic experiences and PTG among Tibetan refugees. Nevertheless, there are few studies linking coping and PTG among Syrian refugees after exposure to traumatic experiences. For instance, a study by Ersahin [24] found that problem-focused coping enhanced PTG, but maladaptive coping such as denial and behavioral disengagement negatively predicted PTG in Syrian refugees. To our knowledge, there has been no study examining the mediator role of coping strategies in the association between past traumatic experiences, post-migration stressors, and PTG among Syrian refugees.

So far, most of the research on PTG among the refugee population has focused on whether positive change occurs or not, rather than the pathways of PTG. There was contradictory evidence regarding the association between multiple trauma exposure and PTG and how this association could be explained by third variables such as coping strategies. Therefore, more empirical evidence in the refugee population is highly demanded to identify certain coping styles to enhance PTG. To address these gaps in the literature and provide preliminary evidence for the pathways of PTG, in this current study we aimed to assess the relationship between multiple trauma exposure and post-migration stressors and PTG among Syrian refugees in Turkey through the possible mediating role of coping strategies. In line with our aim, we proposed the following hypotheses:

1. Post-traumatic growth would be positively associated with past traumatic experiences, but negatively associated with post-migration stressors.
2. Coping strategies would mediate the association between multiple past traumatic experiences and PTG such that past traumatic experiences would be associated with higher use of problem-focused and emotion-focused coping strategies which would predict higher PTG and higher use of maladaptive coping strategies which would predict lower PTG.
3. Coping strategies would mediate the association between post-migration stressors and PTG such that post-migration stressors would be associated with lower use of problem-focused and emotion-focused and higher use of maladaptive coping strategies which would predict lower PTG.

## 2. Materials and Methods

### 2.1. Participants and Procedure

The participants in this study were 528 Syrian refugees, of whom 249 were male (47.2%) and 275 were female (52.1%) with an age range from 18 to 77 years (M = 35.60, SD = 11.65). Data were collected from Hatay and Mardin, the two main Turkish governorates in which Syrian refugees are densely settled near the Turkish Syrian border. Refugees who were under the age of 18, nonnative Arabic speakers and those currently having severe mental disabilities were excluded from the study. We conducted a cross-sectional study which was approved by the ethical committee of Istanbul Sehir University (Institutional Review Board Protocol 25/09). The data collection process took place between December 2019 and February 2020. We used snowballing or chain referral sampling techniques, which are recommended for difficult-to-reach populations [61]. Participants were recruited through a network of Syrian volunteers working at refugee health centers (e.g., doctors and interpreters) and their friends in the cities of Hatay and Mardin. Researchers asked potential participants about their availability for the interviews which took place in the private rooms of a physic and primary health center. Interviews were carried out by native Arabic-speaking interviewers who were informed about the procedures of the study, privacy and safety of participants and also measurement tools by researchers who are experienced in the Clinical Psychology domain. The purpose of the study and its procedure were explained verbally to the participants, and prior to participating they provided their verbal and written consent. After providing informed consent, refugees were interviewed in the Syrian dialects of Arabic for 25–30 min. Refugees did not receive any incentives for their participation in this study.

### 2.2. Measurements

#### 2.2.1. Past Traumatic Experiences

Potential past traumatic experiences were assessed with the Life Event Check List for DSM-5 (LEC-5) [62]. This questionnaire is a self-report 17 item list which uses five answer categories, ranging from “It happened to me” to “not relevant”. Other studies found that LEC has acceptable reliability and variability [63]. There was no standardized scoring for LEC-5, since this questionnaire is aimed at screening potential traumatic events in the whole lifespan. In Sezgin and Punamaki’s [64] article, they decided the scoring as 4 = happening to me, 3 = witnessing, 2 = learning, and 1 = not relevant, in order to assess trauma severity. We averaged items to create target subscales where higher scores displayed greater severity of trauma exposure. As a result of the Confirmatory Factor Analysis (CFA), there were three subtypes of traumatic experiences, which are Natural disasters and accident, Interpersonal violence, and Life-threatening events and war. In this study, we decided to use this specific scoring system and subscales in order to assess the severity of different types of traumatic experiences. Since the Arabic adaptation of the LEC-5 was unavailable, we translated it into Arabic and then back-translation was conducted. We utilized the Confirmatory Factor Analysis (CFA) to test the originally structured three dimensions of past traumatic experiences—Natural disaster and accident, Interpersonal violence, and Life-threatening Events and War—with the current sample. The model fit the data well, χ2 (106) = 351.366 *p* < 0.001 CFI = 0.92, RMSEA = 0.06 (90% CI = 0.059 ~ 0.075). All the items were significantly loaded to the latent factors. Standardized loadings ranged from 0.34 to 0.71, indicating acceptable loading values. We allowed two item residuals covary in the CFA model as it was conceptually meaningful (conveyed similar meanings) and statistically improved the model fit. We used this higher-order latent factor in the structural equation model. Internal consistency (Cronbach’s Alpha) values were 0.79 for natural disasters and accidents, 0.82 for interpersonal violence, and 0.71 for life-threatening and war, in this current study.

#### 2.2.2. Post-Traumatic Growth

The Arabic version of the Post-Traumatic Growth Inventory (PTGI) [65] was used to measure positive changes resulting from traumatic events. The 21-item self-report questionnaire consists of four subscales which are the sense of change and appreciation of life, new opportunities, self-reliance, and ability to express emotion scoring on a 6 point Likert ranging from 0 = “I did not experience this change as a result of my crisis” to 5 = “I experienced this change to a very great degree as a result of my crisis”. We averaged items to create target subscales where higher scores showed higher levels of PTG. Arabic version of the PTGI scale had acceptable internal consistency with Cronbach alpha of 0.94. Recent studies indicated some inconsistency about factor structure as opposed to the five-factor solution found in the original version of the scale. In this sense, some studies suggested that three-factor solutions are more suitable for the conflict-affected population [66]. Therefore, we utilized three-factor structures which were: relating to others, spiritual change, and changes in self. Internal consistency (Cronbach’s Alpha) values were 0.84 for relating to others, 0.90 for changes in self, and 0.78 for spiritual change, in this current study.

#### 2.2.3. Coping Strategies

The 53-item Arabic version of the Coping Scale (COPE) [48] was used to assess responses of participants to stressful life events on a four-point scale ranging from 1 (I usually do not do this at all) to 4 (I usually do this a lot). The original COPE scale [43] included 14 subscales consisting of only two items each. However, the Arabic version of the COPE included three-factor solutions which were problem-focused coping (namely active coping, planning, suppression of competing activities, restraint coping, seeking of instrumental social support), emotion-focused coping (namely seeking of emotional social support, positive reinterpretation, acceptance, turning to religion) and maladaptive coping (namely denial, behavioral and mental disengagement, disengagement by using alcohol or drugs). We averaged items to create target subscales where higher scores indicated higher levels of construct. Arabic version of the COPE had an acceptable internal consistency value with 0.85 for problem-focused coping, 0.82 for emotion-focused coping, and 0.72 for maladaptive coping. In the current study, we found that internal consistency (Cronbach’s Alpha) values were 0.93 for problem-focused coping, 0.88 for emotion-focused coping, and 0.87 for maladaptive coping.

#### 2.2.4. Post-Migration Stressors

The Arabic version of the Post-migration Living Difficulties Scale (PMLD) [12] was used to assess the extent to which post-migration challenges had been a problem for the refugees over the past 12 months. This 17-item scale is rated on a 5-point scale (0 = not a problem to 4 = very serious problem). We averaged items to create a target subscale where higher scores indicated a higher severity of post-migration difficulties. The Arabic version of PMLD included only one subscale, which was integration difficulties consisting of 11 indicators. Those indicators encapsulated the communication difficulties, difficulties with work, worries about not receiving medical treatment, economic problems, difficulties finding housing, social isolation, and discrimination [67]. Cronbach’s alpha value was 0.72 for this subscale in the original study. In the current study, we found that the internal consistency (Cronbach’s alpha) value was 0.76 for integration problems.

### 2.3. Statistical Analyses

Analyses were conducted by using the SPSS 23.0 program (IBM SPSS Statistics for Windows, version 23.0; IBM: Armonk, NY, USA, 2015) [68] and the Mplus 8.4, (Muthén & Muthén M plus program for Windows, version 8.4; Muthén & Muthén: Los Angeles, CA, USA, 2019) [69] program. We tested normality assumptions for each construct by using skewness (|3|) and kurtosis (|8|) criteria [70]. Little’s test of missing completely at random (MCAR), χ2 (24101) = 26,572, *p* < 0.001., suggested that data were missing not completely at random. We accounted for missing data by using a full information maximum likelihood estimation with robust standard errors (FIML) to prevent sample size reduction and subsequent loss of statistical power [71]. We presented standardized coefficients in the structural equation models to report effects sizes [72]. We also tested multivariate outliers by calculating a Mahalanobis distance value χ2 (128) = 183,186 *p* < 0.001, which led removal of 12 participants, the remaining 528 participants were used in further analyses.

We followed a two-step approach in our analyses: first, we tested a measurement model to create predetermined latent variables of post-traumatic growth, post-migration stressors, and traumatic experiences. Coping strategies have predetermined three observed variables: problem-focused, emotion-focused and maladaptive. Although post-migration distress consisted of a single observed indicator, we employed an external measurement reliability approach to account for measurement error for the structured variable in the model [73,74]. We used the reliability coefficient to specify error variance in the factor loading (i.e., θε = Var (1-ρ_(y)) = 0.796 (1–0.72) = 0.23, [73]). Second, we tested the structural model. We utilized top-down model building where we included all possible covariates in the model and then removed the non-significant ones as one at a time by considering the model fit improvement [73]. In each model, age, sex, and length of stay in Turkey were considered as covariates since previous studies concluded that those variables might have an effect on an individual’s experience of traumatic events [47], post-migration stressors [75], and also PTG [76]. In the mediation models, we tested the significance of the indirect effects by using the bootstrapping technique (1000 resampling) with 95% confidence intervals [77]. Following this, model fit indices were used to test model accuracy: comparative fit index (CFI) > 0.90, root mean square error of approximation (RMSEA) < 0.08, and standardized root mean square residual (SRMR) < 0.08 [70,78].

## 3. Results

### 3.1. Preliminary Analyses

As shown in Table 1, results from the bivariate correlation analyses (Pearson’s Correlation) showed participant’s age weakly negatively associated with PTG in self (*r* = −0.13) and relation (*r* = −0.08) but showed no association with other demographic variables. PTG in all domains, which are change in self, spiritual, and relation, were found to be significantly–moderately positively correlated with both problem-solving (*r* = 0.46; 0.46; 0.41) and emotion-focused coping style (*r* = 0.36; 0.35; 0.42), respectively. Maladaptive coping styles were positively associated with integration problems (*r* = 0.23). Additionally, integration problems were significantly positively correlated with PTG such as change in self (*r* = 0.08), relation with others (*r* = 0.21), and spiritual change (*r* = 0.12). While natural disasters and accidents were positively correlated with PTG in all domains, war exposure and life-threatening events were only significantly correlated with PTG in self and spirituality. Interpersonal violence was also significantly and positively related with only PTG in relation. See Table S1 (Supplementary Material) for complete descriptive statistics and bivariate correlations among study variables.

### 3.2. Direct and Indirect Associations

Results from the measurement model showed an acceptable fit to the data, χ2 (12, *n* = 528) = 66,522 *p* < 0.05, RMSEA = 0.093, CFI = 0.96, SRMR = 0.03. Standardized loading values across latent factors ranged from 0.70 to 0.94, indicating acceptable loadings. Overall, results from the measurement model indicated that the theoretically predetermined latent factors could be used for a further structural model.

**Figure 1:**
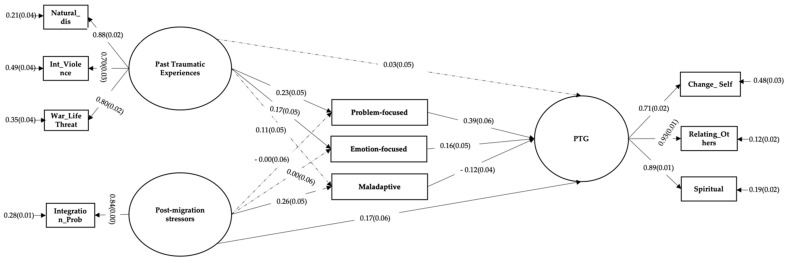
Structural equation model of PTG. Standardized coefficients are presented. Non-significant paths were shown with dotted lines. Natural_dis = Natural disaster and accident, Int_Violence = Interpersonal violence, War_LifeThreat = Life-threatening Events-War, Integration_Prob = Integration problems, Problemfocus= Problem-focused coping, Emotionfocus= Emotion-focused coping, Maladaptive= Maladaptive coping, PTG = Post-traumatic growth.

The final structural model fit the data well, χ2 (43, *n* = 510) = 213.231, *p* < 0.001), CFI = 0.92, RMSEA = 0.08 (90% CI: 0.07–0.10), SRMR = 0.05. As shown in Figure 1, there was a direct effect from traumatic experiences to problem-focused coping (ƴ = 0.23, *p* < 0.001) and emotion-focused coping (ƴ= 0.17, *p* < 0.001). In addition, there was the only a significant direct effect from post migration difficulties to maladaptive coping (ƴ = 0.26, *p* < 0.001). Furthermore, there was a direct effect from problem-focused coping (β= 0.39, *p* < 0.001), emotion-focused coping (β= 0.16, *p* < 0.001) and maladaptive coping (β= −0.12, *p* < 0.001) to PTG. We found a direct effect of post-migration difficulties (ƴ = 0.17), but no direct effect of past traumatic experiences on PTG. The predictors (traumatic experiences, post-migration stressors, and coping strategies) explained 31% of the variance in PTG. The predictors (traumatic experiences, post-migration stressors) explained 5% of the variance in problem-focused, 3% of the variance in emotion focused and 8% of the variance in the maladaptive coping strategies.

We tested the mediating role of coping strategies in the associations between past traumatic experiences, post-migration difficulties, and PTG. Mediation results have shown that the association between past traumatic experiences and PTG was significantly mediated by problem-focused coping (β = 0.12, (95% CI: 0.06–0.20)), and emotion-coping strategies (β = 0.03, (95% CI: 0.01–0.08)). Higher traumatic experiences predicted a higher use of problem-focused and emotion-focused coping strategies, and, in turn, higher use of problem-focused, and emotion-focused coping strategies associated with a higher level of PTG. Furthermore, maladaptive coping strategies significantly mediated the association between post-migration difficulties and PTG (β = −0.03, (95% CI: −0.07–0.01)). Higher post-migration difficulties predicted a higher level of maladaptive coping strategies, and, in turn, higher levels of maladaptive coping strategies associated with a lower level of PTG. See Figure S1 (Supplementary Material) for significant paths in the tested model.

## 4. Discussion

This current study proposed to provide preliminary evidence for the model of PTG, which includes past traumatic experiences, post-migration stressors, and coping strategies among Syrian refugees in Turkey. We assumed that past traumatic experiences and post-migration difficulties would be associated with post-traumatic growth. The relationship between conflict and post-migration related experiences and post-traumatic growth would be mediated by coping strategies. We further postulated that past traumatic experiences would be associated with a higher use of problem and emotion-focused and lower use of maladaptive coping strategies, which would predict higher PTG. Furthermore, post-migration stressors would be associated with a lower use of problem and emotion-focused and higher use of maladaptive coping strategies which would predict lower PTG.

### 4.1. Traumatic Experiences, Post-Migration Stressors and Post-Traumatic Growth

Our results partially confirm the hypothesis concerning the relationship of trauma experiences as well as post-migratory stress and post-traumatic growth. Even though we found that PTG was significantly correlated with different traumatic experiences, in contrast to our expectation and prior literature, we did not find a significant direct effect of past traumatic experiences on PTG regarding the dose–response relationship in our model. These findings might be related to the severity of traumatic experiences due to the fact that the Syrian refugees in our sample were found to be exposed to low levels of past traumatic experiences. Since, as Tedeschi and Calhoun [16] stated, a significant level of trauma is necessary to trigger individual schemas and assumptions which in turn results in positive change. In addition, previous research has reported that refugees who have experienced higher levels of conflict-related traumatic events have reported higher PTG levels [17,18,32,33]. Contrary to our expectations and the literature, post-migration stressors were shown to be positively associated with PTG. Therefore, these findings illustrate that post-migration difficulties might lead to cumulative stress in refugees and, thus, higher stress levels might be related to higher PTG among refugees due to the dose–response relationship [22,31]. Further, discrepancies between the current findings and previous research could be in part due to differences in the nature of samples; therefore, further research is warranted to explore these discrepancies.

In our sample, the mean PTG score was 2.83, which could be considered as a moderate level of PTG [3,14]. Studies with Syrian refugees living in Turkey reported similar levels of PTG [24,25]. This finding is in line with literature indicating that Syrian refugees report some level of the subjective experience of positive psychological changes as a result of highly stressful life events. As the abovementioned studies stated that trauma characteristics are an important predictor of PTG, interestingly our findings showed different results regarding the association between trauma characteristics and PTG. In line with previous studies, our findings indicated the positive association between natural disaster trauma and PTG in all domains [2]. On the other hand, war exposure and life-threatening events were only significantly associated with PTG in self and spirituality. A study by Kira and colleagues [31] reported that while war and life-threatening events were positively associated with internal self-growth and spiritual growth, there was no association between interpersonal traumas and PTG. However, the current study reported that interpersonal violence was also significantly and positively related to only the relation domain of PTG. In this sense, other studies indicated that people who had experienced exposure to sexual abuse and violence reported a positive change in their interpersonal relationships [79,80]. These findings might be related to individual’s increased awareness of who they can build a safe and emotionally close relationship with after traumatic events [81] (p. 12). Even though we did not have any postulation regarding trauma characteristics, our results have revealed that multiply traumatized individuals might have either an impediment or benefit effect for PTG. Therefore, a better understanding of trauma profiles can help to design specific targeted interventions with the identification of potential post-traumatic strengths among refugees [31].

Although we did not have any postulation regarding antecedents of trauma severity among Syrian refugees, we found that the mean of trauma severity ranged 1.62 to 1.97, which could be considered a relatively modest level of trauma among Syrian refugees living in Turkey. However, previous studies reported high rates of traumatic experiences among Syrian refugees [47,82]. These findings might be explained due to the length of stay in the host country, because time elapsed since displacement might undermine the level of trauma exposure with the decay of the stress response [11]. In our study, the average length of stay in Turkey was 5.5 years, which was a relatively longer duration compared to previous studies with Syrian refugees [83]. In addition, we found a negative correlation between traumatic experiences and the duration of stay in Turkey. Further, our sampling method might be another reason to recruit refugees who were exposed to relatively modest trauma. Since we did not test the relationship between trauma severity and other variables, we only speculate possible reasons to explain our sample’s modest level of trauma exposure.

The current study went further to investigate indirect relationships between past traumatic experiences, post-migration stressors, and PTG. Since traumatic experiences themselves do not result in growth, it is important to identify underlying mechanisms that indirectly link past traumatic experiences and post-migration stressors to positive change.

### 4.2. Coping Strategies as a Mediator between Traumatic Experiences, Post-Migration Stressors and, Post-Traumatic Growth

Current results partially supported the hypothesis regarding the mediator role of coping strategies between traumatic experiences and PTG among Syrian refugees. Consistent with other studies, both multiple traumatic experiences and coping were involved directly or indirectly in positive psychological change [18,30]. It is possible to conclude that multiple traumatic experiences might trigger an individual’s certain coping strategies, which determine how an event is perceived and consequently mediates the relationship between trauma and its outcomes [18,59]. In this study, refugees who were exposed to past traumatic experiences were more likely to use higher problem-focused and emotion focused coping strategies and this, in turn, predicts high levels of PTG. In line with the abovementioned studies, past traumatic experiences might catalyze the use of emotion-focused and problem-focused coping strategies [18,51]. Further, these catalyzed emotion-focused, and problem-focused coping strategies may promote PTG. Moreover, these findings are consistent with other studies which have reported problem-focused and emotion-focused coping strategies are more conductive for PTG [18,27,41,60]. It is possible that active coping, focusing on the problem, and managing distress after exposure to traumatic events could promote the experience of post-traumatic growth. Interestingly, we did not find a mediator role of maladaptive coping strategy (i.e., avoidance, denial) in the relationship between past traumatic experiences and PTG. However, a study by Brooks and colleagues [30] revealed that the relationship between multiple traumatic experiences and PTG is explained by avoidance coping among refugees. From that point, the Transactional Model by Lazarus et al. [42,84] could explain the possible reasons for inconsistent findings among refugee studies. According to this model, coping strategies might change based on the demands of specific stressful events in the course of time and thereby, the distinction of adaptive and maladaptive nature of coping strategies might differ due to conflict-related experiences. Moreover, culture is an important factor to determine coping strategies as adaptive or maladaptive [85]. Even though we have conceptualized avoidance and denial coping strategies as maladaptive coping strategies, in our sample, those strategies might have been adaptive considering the effect of culture.

Our hypotheses regarding the role of coping strategies in the relationship between post-migration stressors and PTG were partially supported. We found that post-migration stressors have positively predicted the use of maladaptive coping strategies among Syrian refugees and this, in turn, predicted low levels of PTG. In this sense, social adaptation problems, discrimination, and unemployment might lead to higher cumulative stress, which later depletes adaptive coping resources and triggered maladaptive coping strategies in refugees [47,67,86]. Further, these triggered maladaptive coping strategies may undermine PTG. In line with prior findings, maladaptive coping strategies such as denial and avoidance might have a negative effect on PTG in the refugee sample [76]. On the other hand, we could not find the mediating roles of problem-focused and emotion-focused coping in the relationship between post-migration stressors and PTG. This finding might be explained with the higher level of stressors which might deplete refugee’s effective coping resources such as problem-focused and emotion-focused coping strategies [87]. Furthermore, as we have mentioned above, the beneficial effects of coping strategies are moderated by culture [85] and problem and emotion-focused coping strategies might be less effective in the post-migratory situation of Syrian refugees with resettlement difficulties. Thus, studies regarding PTG, and coping should take into account cultural factors. To our knowledge, there is no study that examines the mediator role of coping in the relationship between post-migration stressors and PTG in the literature. Therefore, further studies are needed to understand this relationship.

Considering the cross-sectional nature of the current study, correlational results could be interpreted the other way around too, which is that refugees’ traumatic experiences and post-migration difficulties may evoke conflictual relationships with their coping strategies. Consistent with Stress Generation Theory [88], previous research showed that coping strategy might also influence risk for subsequent trauma [89], such that adaptive coping strategies predicted a decrease in subsequent trauma exposure [90] whereas maladaptive coping strategies may render people more vulnerable to trauma exposure [91]. Within the refugee population, it is possible to conclude that refugee’s pre-coping strategies might have a determinative role on subsequent trauma exposure. From this perspective, although the current study did not have different time points to investigate concurrent and longitudinal associations between past traumatic experiences, post-migration difficulties, and coping strategies, one could consider that past traumatic experiences, post-migration difficulties and coping strategies may have a bidirectional relationship. Further, post-traumatic growth and coping strategies may have a bidirectional relationship too. Previous studies indicated that post-traumatic growth might promote individuals to learn and adapt to new and more adaptive ways of coping in order to meet the psychological demands of traumatic experiences [90].

### 4.3. Limitations

While the findings of the current study provide us with further underlying mechanisms of PTG in refugee populations, it has several limitations. First, the cross-sectional nature of the study is prohibiting causal interpretations. Thereby, longitudinal studies warrant distinguishing the course and bidirectional nature of PTG [92]. We used a non-random sampling approach to reach people and our sample included only Syrian refugees with temporary protection status living in Hatay and Mardin. Consequently, our findings are not generalizable to those living in different regions and without temporary protection status. Furthermore, our sample was exposed to a low level of traumatic experiences, and we did not utilize any screening tool for the anxiety and depression of the participants. Our purpose was to understand our findings from the perspectives of the general community of refugees; thus, we may also yield different results for refugee samples who have higher stress and poorer mental health. Our measure of traumatic experiences is the LEC questionnaire that has not been adapted and validated among the Syrian refugee population. Finally, self-report measures were implemented in the assessment of PTG, which may be limited in measuring growth without potential errors such as self-report biases, cognitive biases, and recollection distortions due to the retrospective nature of PTG [3]. Further, cognitive indicators of PTG such as rumination and perceived control were unmeasured. Those indicators are important determinants in the theoretical model of PTG [26] and also in previous studies [30,93]. Therefore, further studies may include the cognitive processes to complete a clear picture of PTG in Syrian refugees.

## 5. Conclusions

This study investigated how refugees’ past traumatic experiences and current resettlement related stressors are associated with PTG and identified the mediation of coping strategies in PTG. Findings showed that coping strategies had a determinative role on PTG in Syrian refugees and, thereby, it is essential to develop psychosocial and psychotherapy interventions to enhance coping resources for refugees after conflict-related traumatic events. Based on our findings, those interventions should be aimed to promote problem-focused and emotion-focused coping resources and to diminish the use of maladaptive coping among Syrian refugees. In addition, novel approaches dealing with coping such as neuroeducation/neurodidactics [94] might help refugees to learn the adaptive ways of coping to handle multiple stressors, especially for the increased stress of the COVID-19 pandemic and confinement [95]. Therefore, future studies should be taken into account for the aspect of the COVID-19 pandemic and novel approaches for the coping strategies in refugee mental health. Further, post-migration stressors were found to be important indicators of refugee mental health. Thus, future studies should explore the role of post-migration stressors on PTG in refugees.

## Figures and Tables

**Table 1:** Descriptive statistics and bivariate correlations among study variables.

Variable	1	2	3	4	5	6	7	8	9	10	11	12	13
1. PTG-self	-												
2. PTG-spiritual	0.84 **	-											
3. PTG-relation	0.66 **	0.62 **	-										
4. Natural_dis	0.14 **	0.19 **	0.13 **	-									
5. Int_Violence	0.02	0.04	0.12 **	0.62 **	-								
6. War_lifethreat	0.13 **	0.19 **	0.08	0.71 **	0.57 **	-							
7. Integration prob	0.08 *	0.21 **	0.12 **	0.22 **	0.19 **	0.17 **	-						
8. Problemfocus	0.46 **	0.46 **	0.41 **	0.21 **	0.16 **	0.22 **	0.04	-					
9. Emotionfocus	0.36 **	0.35 **	0.42 **	0.17 **	0.12 **	0.15 **	0.03	0.70 **	-				
10. Maladaptive	0.01	0.02	0.20 **	0.16 **	0.19 **	0.05	0.23 **	0.17 **	0.27 **	-			
11. Age	−0.13 **	−0.08 *	0.02	0.02	0.06	0.02	0.04	−0.01	0.06	−0.04	-		
12. LOS	0.01	−0.08	0.07	−0.14 **	−0.03	−0.15 **	−0.04	0.0	0.02	0.0	0.08 *	-	
13. Sex (1:F 2:M)	0.07	0.0	0.12 **	−0.12 **	−0.17 **	−0.08	0.0	0.0	0.01	−0.04	−0.20 **	−0.09 *	-
*n*	528	528	528	528	528	528	528	528	528	528	525	515	
M	3.05	3.05	2.39	1.97	1.62	1.93	1.78	2.97	3.01	2.19	35.6	65.98	
SD	1.14	1.11	1.16	0.68	0.67	0.75	0.89	0.61	0.5	0.64	11.65	24.04	
Range	0–5	0–5	0–5	1–4	1–4	1–4	0–4	1–4	1–4	1–4	18–77	2–144	
Skewness	0.02	−0.47	−0.43	0.51	1.12	0.44	0.16	−0.47	−0.58	0.07	0.83	−0.154	
Kurtosis	−0.66	−0.23	−0.47	−0.24	0.98	−0.77	−0.44	0.11	0.69	−0.76	0.27	−0.118	

Note. * *p* < 0.05, ** *p* < 0.001. PTG = Post-traumatic growth, Natural_dis = Natural disaster and accident, Int_Violence = Interpersonal violence, War_LifeThreat = Life-threatening Events-War, Integration Prob = Integration problems, Problemfocus= Problem-focused coping, Emotionfocus= Emotion-focused coping, Maladaptive= Maladaptive coping, LOS = Length of stay, F= Female, M= Male.

## Data Availability

The datasets generated during and/or analyzed during the current study are available from the corresponding author on reasonable request.

## Supplementary Materials

The following are available online at https://www.mdpi.com/article/10.3390/ijerph18168829/s1, Figure S1: Structural equation model of PTG., Table S1: Descriptive statistics and bivariate correlations among study variables.
