# Supplementary material for: The Role of Coping Strategies in Post-Traumatic Growth among Syrian Refugees: A Structural Equation Model
Source: Int J Environ Res Public Health. 2021 Aug 21;18(16):8829. doi: 10.3390/ijerph18168829 (PMC8394351; doi:10.3390/ijerph18168829)
Supplement: Supplementary file 1 [file ijerph-18-08829-s001.zip › Table S1.pdf]

Table S1. Descriptive statistics and bivariate correlations among study variables.

Variable	1	2	3	4	5	6	7	8	9	10	11	12	13
1. PTG-self	-												
2. PTG-spiritual	0.84 **	-											
3. PTG-relation	0.66 **	0.62 **	-										
4. Natural_dis	0.14 **	0.19 **	0.13 **	-									
5. Int_Violence	0.02	0.04	0.12 **	0.62 **	-								
6. War_lifethreat	0.13 **	0.19 **	0.08	0.71 **	0.57 **	-							
7. Integration prob	0.08 *	0.21 **	0.12 **	0.22 **	0.19 **	0.17 **	-						
8. Problemfocus	0.46 **	0.46 **	0.41 **	0.21 **	0.16 **	0.22 **	0.04	-					
9. Emotionfocus	0.36 **	0.35 **	0.42 **	0.17 **	0.12 **	0.15 **	0.03	0.70 **	-				
10. Maladaptive	0.01	0.02	0.20 **	0.16 **	0.19 **	0.05	0.23 **	0.17 **	0.27 **	-			
11. Age	−0.13 **	−0.08 *	0.02	0.02	0.06	0.02	0.04	−0.01	0.06	−0.04	-		
12. LOS	0.01	−0.08	0.07	−0.14 **	−0.03	−0.15 **	−0.04	0.0	0.02	0.0	0.08 *	-	
13. Sex (1:F 2:M)	0.07	0.0	0.12 **	−0.12 **	−0.17 **	−0.08	0.0	0.0	0.01	−0.04	−0.20 **	−0.09 *	-
n	528	528	528	528	528	528	528	528	528	528	525	515	
M	3.05	3.05	2.39	1.97	1.62	1.93	1.78	2.97	3.01	2.19	35.6	65.98	
SD	1.14	1.11	1.16	0.68	0.67	0.75	0.89	0.61	0.5	0.64	11.65	24.04	
Range	0–5	0–5	0–5	1–4	1–4	1–4	0–4	1–4	1–4	1–4	18–77	2–144	
Skewness	0.02	−0.47	−0.43	0.51	1.12	0.44	0.16	−0.47	−0.58	0.07	0.83	−0.154	
Kurtosis	−0.66	−0.23	−0.47	−0.24	0.98	−0.77	−0.44	0.11	0.69	−0.76	0.27	−0.118	

Note. * $p < 0.05$, ** $p < 0.001$. PTG = Post-traumatic growth, Natural_dis = Natural disaster and accident, Int_Violence = Interpersonal violence, War_LifeThreat = Life-threatening Events-War, Integration Prob = Integration problems, Problemfocus = Problem-focused coping, Emotionfocus = Emotion-focused coping, Maladaptive = Maladaptive coping, LOS = Length of stay, F= Female, M = Male.
