# Supplementary material for: The Role of Coping Strategies in Post-Traumatic Growth among Syrian Refugees: A Structural Equation Model
Source: Int J Environ Res Public Health. 2021 Aug 21;18(16):8829. doi: 10.3390/ijerph18168829 (PMC8394351; doi:10.3390/ijerph18168829)
Supplement: Supplementary file 1 [file ijerph-18-08829-s001.zip › Figure S1.pdf]

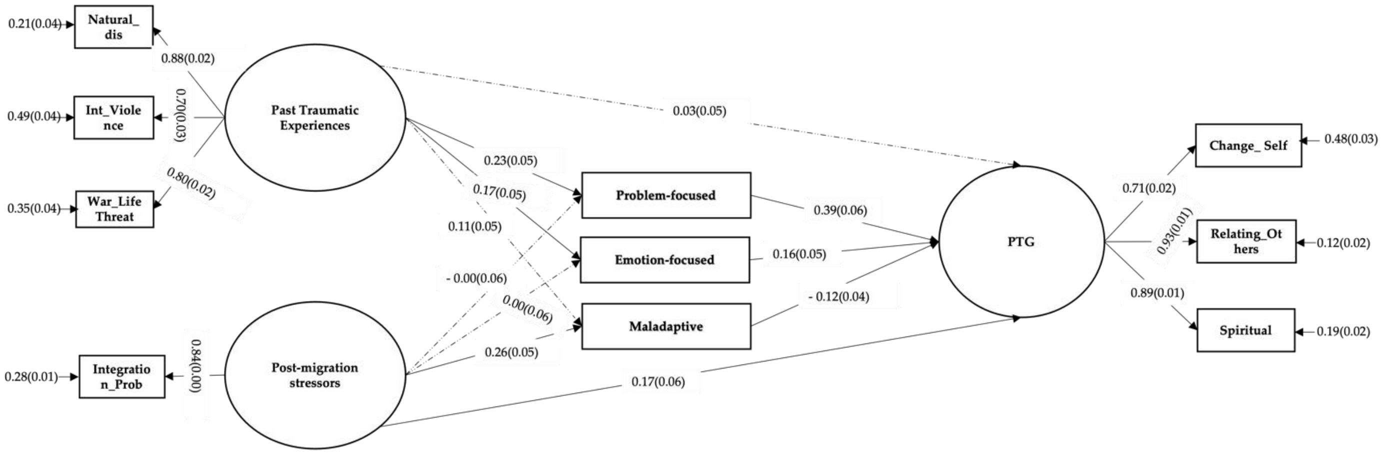

Figure S1. Structural equation model of PTG. Standardized coefficients are presented. Non-significant paths were shown with dotted lines. Natural_dis = Natural disaster and accident, Int_Violence = Interpersonal violence, War_LifeThreat = Life-threatening Events-War, Integration_Prob = Integration problems, Problemfocus= Problem-focused coping, Emotionfocus= Emotion-focused coping, Maladaptive= Maladaptive coping, PTG = Post-traumatic growth.
